# Automatic Registration of Footsteps in Contact Regions for Reactive Agility Training in Sports

**DOI:** 10.3390/s20061709

**Published:** 2020-03-19

**Authors:** Eduardo C. Latorre, Marcos D. Zuniga, Enrique Arriaza, Fabian Moya, Christopher Nikulin

**Affiliations:** 1 Electronics Department, Universidad Técnica Federico Santa María, Valparaíso 2390123, Chile; marcos.zuniga@usm.cl; 2 Advanced Studies Center, Universidad de Playa Ancha, Valparaíso 2360072, Chile; earriaza@upla.cl; 3 Physical Activity and Sports Research Laboratory, Universidad Arturo Prat, Valparaíso 2360072, Chile; f.moyavergara@gmail.com; 4 Physical Education Pedagogy, Education Faculty, Universidad Viña del Mar, Viña de Mar 2520000, Chile; 5 Product Design Engineering Department, Universidad Técnica Federico Santa María, Valparaíso 2390123, Chile; christopher.nikulin@usm.cl

**Keywords:** step detection, foot tracking, reactive agility evaluation

## Abstract

In collective sports, reactive agility training methodologies allow to evaluate and improve the player performance, being able to consider a mixture of technical, tactical, physical, and psychological abilities, similarly to real game-play situations. In this article, we present a new methodology for reactive agility training (neural training), the technological setup for the methodology, and a new footstep tracking algorithm, as the key element for automating the speed data gathering process, necessary for obtaining the relevant variables of the neural training approach. This new methodology is oriented to accurately measure two of the most relevant variables for reactive agility training: total response time (sprint time) and response correctness, related to a stimuli sequence presented to a player. The stimuli were designed to properly represent realistic competitive conditions for player training, contextualized to soccer. In order to automate the gathering process, a new computer vision based automatic footstep detection algorithm has been integrated to the system. The algorithm combines Kalman Filters, segmentation techniques, and perspective geometry, for obtaining highly precise detections of the moment a relevant footstep occurs in real-time, reaching a precision higher than 97%. Plus, the algorithm does not require any special marker, invasive sensor, or clothing constraint on the player.

## 1. Introduction

Among team sports, soccer is the most popular sport in the world. It is practiced at competitive level massively for both men and women, children and adults, and it is one of the biggest industries worldwide, with a market size of 25 billion euros between 2016 and 2017, just in Europe [1]. As in the majority of team sports, soccer includes a great variety of situations that require explosive displacements in presence of unexpected circumstances. These situations demand the application of changes in pace, rhythm and direction, reactive sprints and stops, jumps, and a variety of slide tackles to reach a determined goal [2], such as evading an opponent, taking a pass position in the field, anticipating an opponent’s pass, getting out of the off-side area, blocking shoots to goal, and so forth.

In particular, change of direction, acceleration, and deceleration are the displacements defining the most important plays in the game. Generally, these plays are given in the most congested zones and under some kind of haste or pressure (time or marker). The player’s ability to execute such movements in a frequent and efficient manner, is a fundamental requirement to practice this and any other team sport. Nevertheless, the differentiation between this resource and any other is demarcated by the capabilities of the player who executes it, having to take adequate decisions at high speed under pressure.

Considering the above, displacement and perceptual-cognitive abilities altogether are the elements of an effective response in the field that have a great influence in the game development and the concept that integrates them is the reactive agility, as Sheppard and Young mentioned in Reference [3]. In fact, the definition of reactive agility states that this corresponds to a “quick movement of all body with changes of speed and direction in response to a stimulus” [2], which distinguishes changes of direction in the movement (CODs), perceptual, and decision making components as it explains Young model in Reference [4].

There are different methods for measuring changes of direction and speed agility of players in team sports, such as 505 Test (505) [5] and Illinois Agility Test (IAT) [6]. Even though both methods are widely researched and commonly used, the 505 test only considers one COD in 180 degrees, while IAT test considers distances that result in fatigue instead of a quick and explosive response. Also, it is important to mention that both methods do not consider the perceptual and decision making components, since they are preconceived, planned, and invariant routines [7]. Therefore, these methods are decontextualised of any usual sport behavior.

In this article, we present a new methodology for cognitive reactive agility training (neural training), the technological setup for flexible deployment of the methodology, and a new footstep tracking algorithm as the key element of the neural training system. This system and the implemented methodology have been primarily contextualized and tested for soccer players, based on the thesis work of Moya [8]. This method utilizes a physical platform with regions of interest delimited by color and form, in which the player must interact in order to respond to visual stimuli presented by a screen located in front of the platform. The stimuli are properly designed to represent real game-play situations, so that the player must be able to physically react with the correct response as quickly as possible. The data associated to the player displacement is captured by a video camera disposed in front of the platform, connected to a software that processes the video frames, and synchronizes the sequence of stimuli in real-time.

Several contributions can be clearly identified in this work: First, this is the first article describing this new methodology for reactive training; it is the first methodology which contextualizes the reactive agility with visual stimuli relate to the specific sport, in the context of collective sports training. Second, the setup has been technically thought to allow an easy deployment and, in the future, extend its applicability to the real field; the whole system works wirelessly in real-time. Third, a new footstep detection algorithm, allowing the automation of the process of registering the physical responses of the player, with high precision, without the need of special markers or invasive sensors. This approach is subject to a pending patent application.

For validating the proposed approach we tested the system in real operation conditions, for three players and three training routines each, comparing the results from manual registration and automatic footstep detection against ground-truth data. Using the automatic footstep detection in contact regions, the system reached a precision higher than 97%, outperforming the manual registration procedure.

This article is structured as follows. Section 2 discusses the most similar approaches in both the state-of-the-art and in the Section 3 we present the new collective sports training methodology and the algorithm that automatically allows to visually obtain highly precise measurements of the footstep of the sportsmen. Then, in Section 4, we present the experiments and results focused on validating the capability of the system on operating automatically. Finally, in Section 5 we presents the conclusions and future work.

## 2. Related Work

### 2.1. Relevant Tools for Sports Training

Many tools have been designed for the evaluation of different variables in sports training. However, in general, these tools measure independent capabilities or abilities, which implies necessarily the decontextualization of the habitual sport behavior, and consequently, they are far away of reactive agility training requirements.

One of the most popular tools is Fitlight^®^ [9], which consists in a set of devices equipped with LED lights that turn on according to a predefined sequence, where the player must turn the lights off by reaching these devices with some limb, depending on the routine and the specific sport. Nevertheless, this tool only is capable of measuring reaction time. Another popular tool is the Vienna Test System Sport^®^ [10], which offers a series of tests for team players, in which movement anticipation measure, information processing velocity, and reaction time tests stand out. Moreover, this tool only aims at measuring team sport player cognitive capabilities.

Besides, Neurotracker^©^ uses 3D-MOT (three-dimensional multiple object tracking), for training peripheral vision, attention, visual information processing speed, and response time to peripheral stimuli improvements [11]. Therefore, in general, the previously mentioned tools are based on visual perception and answer to predefined stimuli [12] or multi-object tracking [13].

On the other hand, displacement speed evaluation and training has been another relevant focus of attention for investigators and designers of technological tools.

SpeedCourt training tool [14], have been thought for measuring the linear displacement speed with changes of directions. However, it corresponds to predefined movements where pure capacity of reaction and displacement are evaluated, without considering decision making factors, which is an essential requirement for reactive agility evaluation and training. Also, it is necessary to mention that these training platforms require a reserved and suitable space to be used, due to sensors disposed on the floor and the need of a special smooth platform for properly detecting the footsteps of players, reducing its capability of being portable or used in the sport field. In this sense, solutions based on the use of other type of sensors can be versatile alternatives, but they require special algorithms for automatic footstep detection.

In this sense, in terms of methodology, the approach presented in this article proposes a new evaluation method allowing to evaluate the cognitive efforts required by motor actions in sports [15]. Understanding that these efforts are regulated by complex systems inside the brain structure jointly acting with the motor responses to ensure the efficiency of the action, it is not admissible to isolate the cognitive from the physical component [16]. Also, to improve the effectiveness of perceptive-cognitive training, the proposed tool has been designed to be easily deployed, considering wireless communications for all its components.

### 2.2. Footstep Detection Approaches

In order to achieve high accuracy in the evaluation, it is not recommendable to rely on manual operators. For this purpose, automatic footstep detection approaches can be integrated. A wide variety of footstep detection algorithms exist based on the use of accelerometers and gyroscopes. In Reference [17], an algorithm is proposed for footstep recognition that uses an accelerometer in a smartphone device. The proposed algorithm is divided in two stages: data acquisition and data analysis. The first phase aims at obtaining the data from the accelerometer and processes it to obtain a segment initialization, while the second stage identifies correlated segments that can be considered as footsteps.

In general, footstep counting methods based on accelerometers tend to overcount, because of their tendency to confuse sensor vertical movements as subject footsteps, counting even when the subject takes an elevator, is in a car travel or intentionally moves the sensor (or smartphone) vertically [18]. Jayalath et al. in Reference [19] present an algorithm for footstep detection using a gyroscope embedded in a smartphone. Given the legs movement while walking has an oscillating behavior, said movement can be identified by monitoring legs angular velocity, positioning the smartphone vertically in the subject pocket. After the signal of interest is acquired, it is filtered because a typical walk can be near of 2 Hz, while running can be near of 4 Hz. The main idea of this approach is to detect crosses in zero velocity threshold within the signal of interest, along with configuring a threshold parameter for each individual, that regulates the peak size necessary for step detection. Also, this algorithm considers a delay time between footsteps, used for diminishing wrong counting of consecutive footsteps.

In the context of computer vision, Wang et al. in Reference [20], present a method for people identification by gait. The method consists in identifying and tracking the subject moving silhouette by segmentation and tracking algorithms. Later, PCA (Principal Component Analysis) is applied to the distance signals between the silhouette centroid to each of the border points for dimensionality reduction. For the classification stage, a similarity measure (STC: Spatial-Temporal Correlation) between input sequences is used, which is synchronized by a frequency and phase analysis of the observed footsteps. For this last phase of footstep frequency analysis, a strategy similar to Reference [21] is used, considering the aspect relation of the mobile silhouette bounding box as a real-time input signal. Then, the background effect is subtracted to calculate the autocorrelation and the first order derivative, and so finding the peak time position (footsteps) of each frame sequence.

Harle et al. [22] propose an algorithm for automatic identification of footstep time and pixel position for athletes in high speed videos. This work considers certain constraints, like a static camera, one single runner being monitored and no real-time limitation for the algorithm. The main strategy of Harle’s algorithm is to accumulate the value of pixels that do not have a significant variation, because foot is the only stationary body part when the subject takes a step. Thereafter, the accumulation resulting zones are evaluated through a threshold that takes the average time of footsteps ground contact. Given that background subtraction problems may exist, one last filtering stage of footstep candidates is considered. The first filter considers a consensus of footstep sampling, that is to say, it assumes that the subject takes footsteps in a regular time period during a sprint and generally in straight line, while the second filter considers a run cycle period, meaning that it is possible to estimate when a footstep ground contact is expected using the runner’s silhouette size obtained in the segmentation stage.

In the latest years, machine learning approaches has become very popular. In particular, in the context of computer vision CNN (Convolutional Neural Network) approaches are the most studied and utilized. In the context of footstep detection, few works can be found, reporting results which are far from the precision needs of a system to be operated in real conditions—in Reference [23] the authors report a classification accuracy of 61.20% and in Reference [24] an accuracy of 87.66% is reported. In both cases, the training bases do not consider occlusion situations, which are very frequent in the sequences captured for the methodology presented in this article.

The proposed footstep detection approach is image-based and automated using computer vision techniques. The system transmits the information from a camera attached to a small single-board computer to a server, which performs the processing. This way, there is no need of a fixed space for deployment, allowing the versatility of the system to be used in different spaces as gyms and different sectors of a sport field (considering similar training space dimensions), without the need of having specialized designed rooms for the training routines, as SpeedCourt case [14]. Besides, the approach is non-invasive as no on-player sensor is required and take no footstep regularity assumptions as the player movement and reactive and unpredictable.

## 3. Materials and Methods

In collective sports, speed is one of the key aspects for a successful performance. The evaluation of speed can be divided in two fundamental aspects: as motor, and as motor-cognitive attribute. For a collective sport player, it is the data processing speed of the variables in the environment, the aspect that establishes the most significant difference between success and failures [8].

In order to evaluate the speed as a basic motor attribute, the player is considered as a mere executor of motor actions. Therefore, the evaluation of this aspect of speed quantifies how long does it take the player to execute a previously determined movement, as it is proposed in 505 Test and IAT methodologies, previously described. For evaluating speed from the motor-cognitive point of view, the player is recognized as an executor of movements conditioned by a prior data processing phase, and the time on reacting to a stimulus, and executing the response is measured, as described by Sheppard et al. [3]. In this sense, the concept which unifies speed training as a motor-cognitive paradigm, is the reactive agility training. Several works can be found showing that reactive agility training is useful for identifying the most skillful players, as in Farrow et al. [25].

In general, the methodologies for reactive speed training consist in presenting a virtual game-play situation, where the player has to analyze a tactical situation in the context of the corresponding sport. Based on her/his experience, the player must decide the right response, generating a displacement coherent to this response. Then, the reaction time, total response time, and correctness of response variables are quantified (Bullock et al. [26]).

In this article we focus on the total response time and correctness of response variables, through a new methodology for reactive speed training, which has been developed jointly with the presented algorithm, in order to be able of obtaining these variables in an automated way.

The experiment consists in a reactive agility test that measures physical and cognitive abilities in team sport players. In particular, for the present work, the test is designed for soccer players. The model consists in a platform of nine red square targets corresponding to the indicative zone positions to which the athlete must move, according to the response that the player decides for a received stimulus.

**Figure 1:**
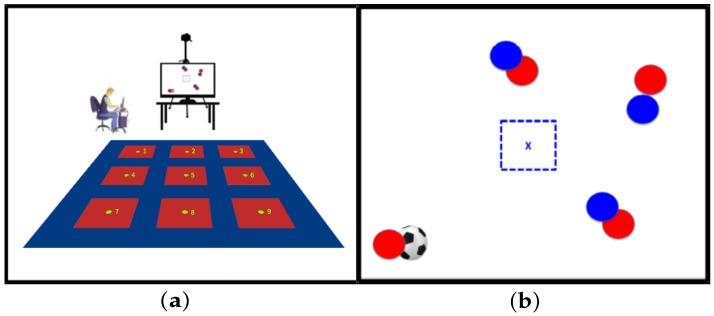
Training platform and visual stimulus presented to a team sport player. (**a**) Training platform with nine red square targets. (**b**) Visual stimulus of soccer tactical situation [8].

Each target center is placed at two meters of distance between each other, as depicted in Figure 1a. A LED screen is placed in front of the platform for a sequence of visual stimuli that simulates soccer tactical situations to which the player must respond (see Figure 1a).

The test begins with the player standing on the platform’s red-square central target, waiting for the first stimulus. Given this first tactical situation stimulus, the player must identify her/his role (defending or attacking), and infer the right response, moving accordingly to the zone center in a peripheral red-square target, assuming a passing reception or marking position according to the identified role, and quickly return to the starting point to activate the next stimulus (see Figure 1b).

The player movement on the platform is captured by a video camera placed in front of the platform (see Figure 1). The camera position is 6.5 [m] in front of the central target 5, disposed in the platform middle, and at a height of 3.5 [m]. Camera max resolution is 1280 × 720 at 30 fps. The video frames are transmitted to a software that processes the acquired video and synchronizes the visual stimuli sequence in real-time. The reactive agility measure is obtained in function of the efficiency on decision making, plus the physical displacement, considered from the time the player reacts to a tactical situation on screen, that it to say, the total elapsed time between the stimulus presentation and the time the right target is reached.

**Figure 2:**
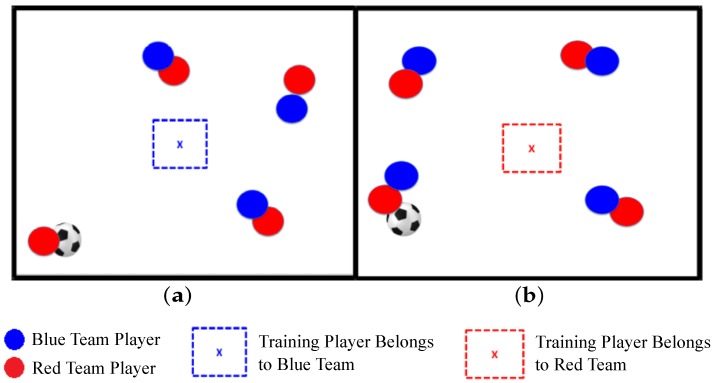
Visual stimuli examples of soccer marking and passing tactical situations. (**a**) Visual stimulus of defensive tactical situation. (**b**) Visual stimulus of offensive tactical situation [8].

It is important to say that the target zones are not explicit in the image. They must be inferred through interpretation of the tactical situation, where the circles represent other players in the field, and the color of the dashed square in the screen center represents the team to which the player who is training belongs (see Figure 2).

**Figure 3:**
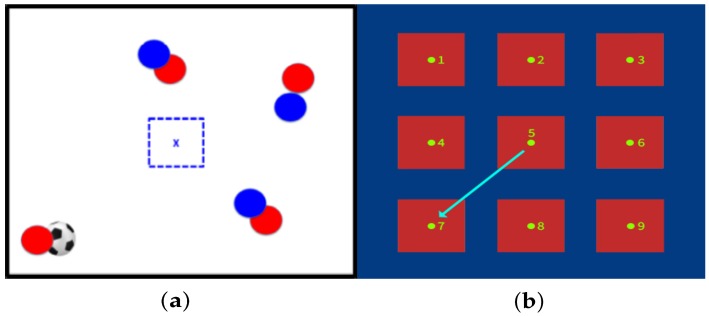
Example of soccer tactical situation and the expected response. (**a**) Defensive tactical situation marks red player with ball possession. (**b**) Response is running to target 7 on the platform (assuming marking position).

See, for example, Figure 3 which explains a defensive soccer tactical situation stimulus to be interpreted. The image in Figure 3a represents a defensive tactical situation in which the player belongs to the blue team, given the blue dashed center square. Then, the right response if the player is to assume a marking position, near the red player with ball possession. Therefore, the player must go to target 7 in the training platform as expected response, as shown in Figure 3b.

**Figure 4:**
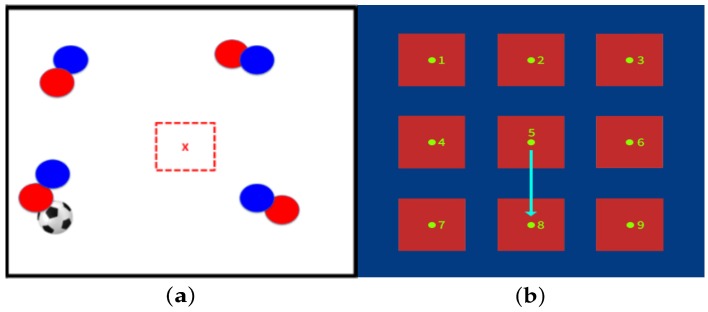
Example of soccer tactical situation and the expected response. (**a**) Offensive tactical situation, go to an uncovered pass position. (**b**) Response is running to target 8 on the platform.

In Figure 4 an offensive soccer tactical situation stimulus to be interpreted is presented. Figure 4a represents an offensive tactical situation in which the training player belongs to the red team, given the red dashed center square, and has to run to an uncovered position to receive a pass. Therefore, the expected response is that the player must go to target 8 in the training platform, as shown in Figure 4b.

### 3.1. Footstep Detection Algorithm

In this section, we present our proposal of a new footstep detection algorithm capable of recognizing with high precision the exact moment when a player steps in the target contact region. The application context is the neural training approach described in previous section, where a precise response is required as soon as possible, because the proposed system must be immediately responsive to the interaction of the player’s feet contact to the surface in specific zones in a structured field.

**Figure 5:**
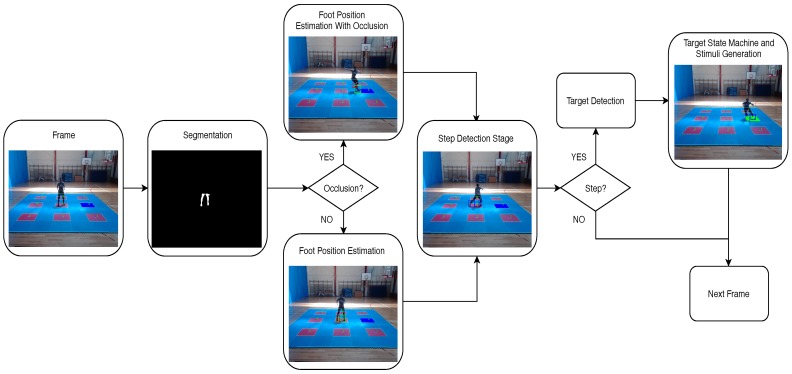
Algorithm stages diagram.

The proposed algorithm for automatic footstep detection can be divided in four stages (see Figure 5). Initially, a segmentation stage is applied to remove the static background for first isolating the region of interest (ROI) of the player silhouette, and then reducing the ROI to the lower body parts (player feet). Next, a discrimination phase is applied to the ROI for determining if the feet are separated or a self-occlusion situation is occurring (feet crossing situation); based on this selection, the feet positioning measurement is obtained. Then, a Kalman filter stage is implemented for feet positioning prediction and the model is adjusted according to the previously obtained feet positioning measurements. Next, a footstep detection (ground contact) stage is performed, considering the measured positions and Kalman filter predictions. Finally, the algorithm inspects if the footstep positions correspond to a platform red-square center target with some tolerance degree, and provides feedback to the training system if this occurs. Here under, each stage is described in detail:

#### 3.1.1. Background Subtraction and Calibration

**Figure 6:**
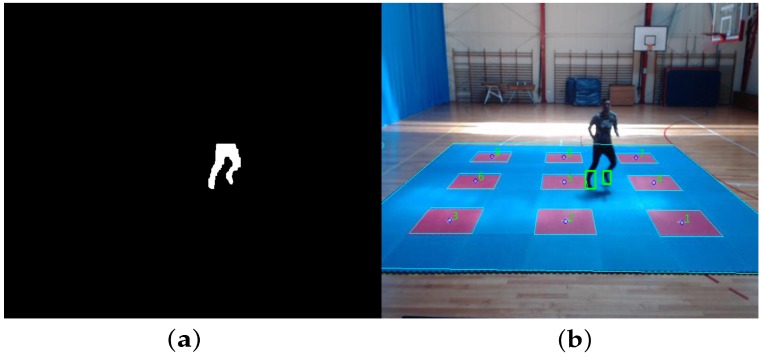
Background subtraction, region of interest (ROI) selection and foot position measure. (**a**) Background subtraction. (**b**) Feet selection.

**Figure 7:**
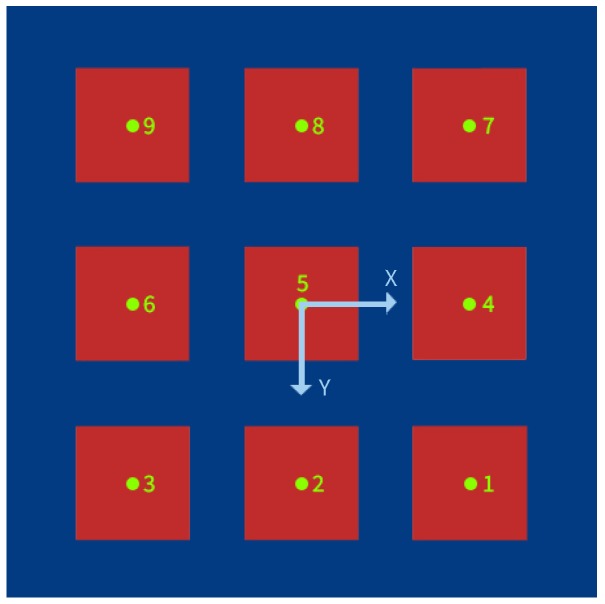
Coordinate system, floor level plane centered on target 5.

The first step of the proposed algorithm aims to subtract the non-moving regions. The method considers a static camera observing a static scene with one moving object (person). Therefore, a valid assumption is that the background image has a regular behavior that can be described by a statistical model as Gaussian Mixture Model (GMM) [27]. Since the true interest region is related with the subject feet, another consideration is to apply a scenario mask for only keeping the silhouette part which lies inside the training platform (see Figure 6a). For this purpose, an homography matrix of the platform plane is obtained in a prior configuration stage, through a calibration process using the real training platform points in meters, and considering the center of the central target (target 5) as the origin (0,0) of the platform, defining the coordinate system as a floor level plane centered on target 5 with a positive Y axis in the direction of the camera position and a positive X axis towards the right side of the camera (see Figure 7).

Therefore, calibrating the homography matrix also allows to gate every target position in the platform at pixel level (polygon enclosing each of the nine target regions), and the pixel position registered after the footstep detection phase can then be translated into a real scene position, as shown in Figure 6b.

Also, due to the training method design, the player always starts standing in target 5 looking the initial stimulus. Therefore, the initial value for the bounding boxes width and height and their position is calculated from the foreground mask obtained from the segmentation stage. That is to say, the bottom 20% of the subject’s silhouette is taken as initial bounding box, for positioning left and right feet, and obtaining the initial measurement.

#### 3.1.2. Kalman Filter

The Kalman filter is a linear estimator that predicts and filters linear system states. It is an attractive tool since it is the optimal linear estimator that minimizes the estimation error variance and can be implemented recursively [28].

In this work, a feet tracking system is considered, where x(k) is the state vector of dimension 6, for a foot, that can be expressed in Equation (1).

(1) $$x\left(k\right)=[x\left(k\right),\;y\left(k\right),\;v_{x}\left(k\right),\;v_{y}\left(k\right),\;w\left(k\right),\;h\left(k\right)],$$

where x(k), y(k) are the pixel position for the foot bounding box, $v_{x}\left(k\right)$ and $v_{y}\left(k\right)$ are the foot bounding box speed, and w(k), h(k) are its width and height, respectively. Then, the process and measure equations for a foot are given by:

(2) x(k)=Ax(k-1)+n(k),

(3) z(k)=Hx(k)+e(k),

where n(k) is the process Gaussian noise, and e(k) is the measurement Gaussian noise, whereas *k* represents discrete time instants associated to each video frame. The main goal is to estimate the x(k+1) state given the measures z(k) and the previous state information x(k-1). For this purpose, the transition and measurement matrices are defined, as Li et al. in Reference [22], where Δt is the sample period:

(4) $$A=\left[\begin{array}{cccccc} 1 & 0 & \Delta t & 0 & 0 & 0\\ 0 & 1 & 0 & \Delta t & 0 & 0\\ 0 & 0 & 1 & 0 & 0 & 0\\ 0 & 0 & 0 & 1 & 0 & 0\\ 0 & 0 & 0 & 0 & 1 & 0\\ 0 & 0 & 0 & 0 & 0 & 1 \end{array}\right]$$

(5) $$H=\left[\begin{array}{cccccc} 1 & 0 & 0 & 0 & 0 & 0\\ 0 & 1 & 0 & 0 & 0 & 0\\ 0 & 0 & 0 & 0 & 1 & 0\\ 0 & 0 & 0 & 0 & 0 & 1 \end{array}\right]$$

With the previous model, the process and measurement equations, a Kalman filter can be used for tracking both left and right feet, associating one filter for each foot.

#### 3.1.3. Step Detection Stage

First of all, a series of player training videos were analyzed, to establish assumptions that will help the footstep detection stage, as Jayalath et al. in Reference [19]. It was observed that a player footstep takes between 2–4 video frames in foot-down and toe-off events [22], so that the footstep position inside the platform is considerably static in this time period. Also, it can be deduced that, in a training routine, the soccer player attacks the target and then initiates a change of direction (COD) displacement back to the platform center, so every time that the player steps into a target, the static condition of her/his foot occurs.

**Figure 8:**
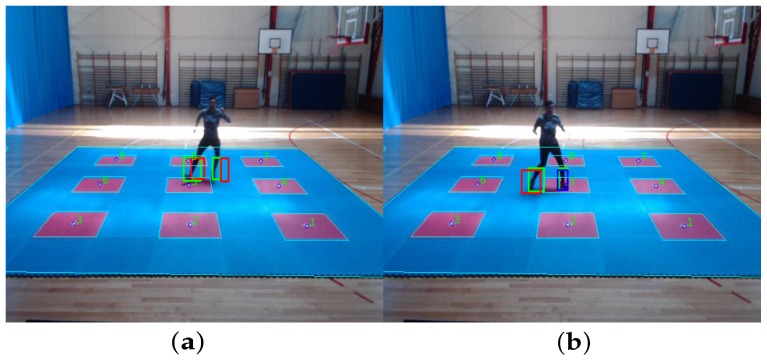
Kalman filter prediction and footstep detection stage for reinitialization and positive footstep detection. (**a**) Kalman filter prediction and reinitialization for both foots. (**b**) Kalman filter prediction and left footstep detection.

Given the previous assumptions, the footstep detection stage starts by calculating the euclidean distance between the Kalman predicted position state and the measurement of the foot position for the current frame. If the measured distance is higher than a threshold, this means that probably the foot is moving, so it is unlikely to correspond to a step (see Figure 8a), as Harle et al. in Reference [22]. In this case, the Kalman filter is reset with the previous measurement. In the case the foot starts decelerating, the distance would be lower than a threshold, in which case the filter is updated with the current measurement. After the filter is updated, a second threshold is evaluated, checking if the prediction is close enough to the measurement, in which case it is classified as a step.

From Figure 8b, green bounding boxes show the current frame foot position measurement, red bounding boxes represent the result of Kalman filter prediction, and blue bounding boxes represent the positive footstep detection stage (blue boxes are a footstep detection for the related foot).

#### 3.1.4. Footstep in Contact Region

In this section the algorithm aims to determine if a detected footstep is a positive match according to the current stimulus from the active training session, if an erroneous match occurred due to a mistakenly reached target, or just a footstep out of any red-square target in the training platform.

In order to determine a target matching situation, this stage takes the previously detected footstep positioning data, transforms the corresponding image points to platform coordinates (using homography transform), and measures the distance to every target. If the distance between a detected footstep and a target center is less than an fixed threshold of 30 [cm], it is considered that the footstep reached the target; on the contrary, no matching event is triggered. This threshold, indicating the average size of support surfaces areas in contact regions, has been empirically established from pilot tests.

**Figure 9:**
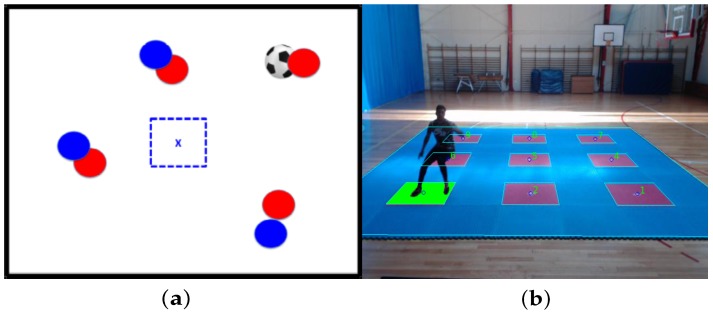
Correct target match according to visual stimulus. (**a**) Visual tactical soccer situation. (**b**) Matched target according to the stimulus.

**Figure 10:**
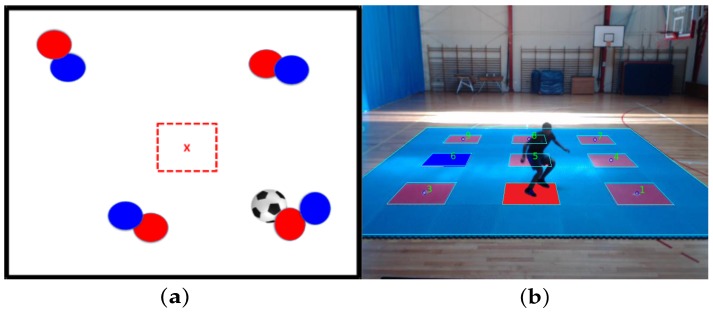
Mistaken target match according to visual stimulus. (**a**) Visual tactical soccer situation. (**b**) Mistakenly matched target according to the stimulus.

As shown in Figure 9, a positive matching situation is when the player reaches the same red-square target as the target to be inferred from the stimulus. In this case, target 3 should be inferred and the player reaches target 3, so the positive matching zone is colored green. On the contrary, as shown in Figure 10, a negative matching situation is when the player reaches a mistaken target according to the stimulus. In this example, the player reaches target 2, but the proposed stimulus target is 6, so the negative matching zone is colored red and the actual target zone is colored blue.

**Figure 11:**
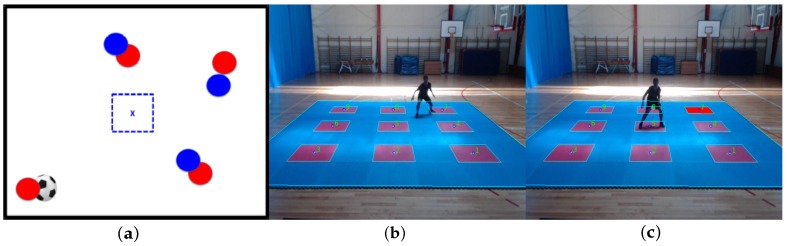
No target situation and no target situation detected in platform central position. (**a**) Visual tactical soccer situation. (**b**) No reaching target situation. (**c**) No reached target situation detected (back in center).

Finally, a footstep out of the target zone situation is described in Figure 11. Figure 11b presents a player attack situation to target 7, but the footstep detection is not close enough to the target center, so no matching is triggered. This situation is registered when the player comes back to the platform center; in this case, the missed target zone is colored red as shown in Figure 11c.

In order to analyze the detection efficiency on automatic footstep registration of the proposed algorithm, next section presents the developed ground-truth, the experiments, and results of the approach.

## 4. Evaluation and Results

This section describes experimental results of the proposed algorithm for automatic registration of footsteps in contact regions. The algorithm was implemented in C++ language using OpenCV libraries, and the experiment was performed in a Core i5 3.5 GHz processor computer. An analysis of the precision, sensitivity, and specificity of the approach is provided, considering different segmentation thresholds for representing possible illumination changes, effect of noise, and different occlusion situations in a non-controlled training field (e.g., soccer field).

Also a comparison analysis formanual and automatic registration is presented. Manual measurement registration has been performed using an application (NeuroCoach) specifically built for this purpose, which is able of recording videos and manually register times and frames in which the player steps into a contact zone.

**Figure 12:**
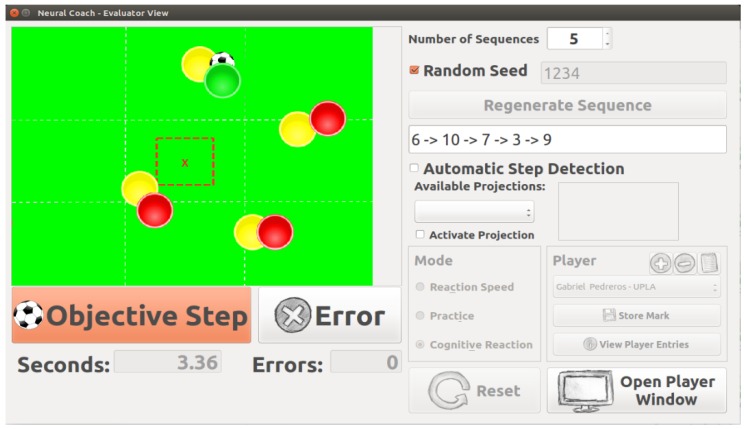
Human-Machine Interface on NeuroCoach application.

The manual time registration process was done using human-machine interface on NeuroCoach application (see Figure 12). Times and frames were stored by pressing keyboard shortcuts, while the operator watched the player reaching a target, and running back to the platform center. Four subjects performed preliminary tests as operator, and for the comparative tests of this article the most experienced operator was considered. On the other hand, the automatic registration was done utilizing the proposed footstep detection algorithm, storing the corresponding frame and time when the player steps in a contact region.

### 4.1. Training Dataset

The players participating as test subjects for the training experiment belong to the Sciences of Sports and Physical Activities Faculty of Playa Ancha University, Chile. The dataset utilized to evaluate the algorithm performance consists in three training videos for three soccer players; that is to say, nine recorded videos at 20 fps sample frequency and 640 × 480 resolution, that is a total of 3807 frames, suited for an automatic training tool.

Training videos were recorded considering natural light. Therefore, the sequences contain noise due to changes of illumination caused by people walking in front of the gym lateral light entrance. Changes of light are also produced by passing clouds (gym with skylight), inducing different conditions for player shadows.

Training videos were recorded independently for each player, considering random stimuli training sequences, with the purpose of running the experiment like a real training test. Along with the video sequence, manual registration of times for footsteps in contact regions was performed, using the manual registration option by a human operator, for comparing the manual with the automatic registration.

### 4.2. Ground-Truth and Evaluation

For performing the evaluation, ground-truth information was generated offline for each video sequence, considering as annotated time of footstep detection the frame number in which the player steps into a contact region, multiplied by the time between frames. The correctness on the response performed by the player is also stored. The comparison between ground-truth, manual, and automatic registration is done by comparing the frame times and targets reached by the soccer players along the training routine.

For a True Positive (TP) detection, the condition is that the automatically registered target coincides with the ground-truth target in a time difference of at most 250 [ms] [29] (less or equal to 5 frames of difference). A False Positive (FP) registration occurs when the algorithm detects a footstep in a contact region, which is not in the ground-truth, while a False Negative (FN) is when the algorithm does not recognize an event that is in the ground-truth. Finally, a True Negative (TN) situation occurs when the player does not reach any target for the particular play (or stimulus) and the algorithm does not trigger any event associated to the same play (or stimulus).

### 4.3. Experimental Results

**Figure 13:**
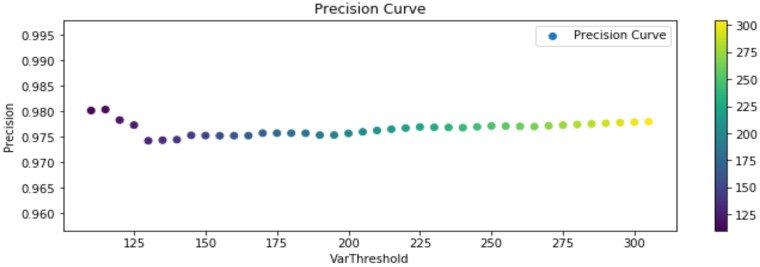
Precision True Positive (TP)/(TP + False Positive (FP)) results.

**Figure 14:**
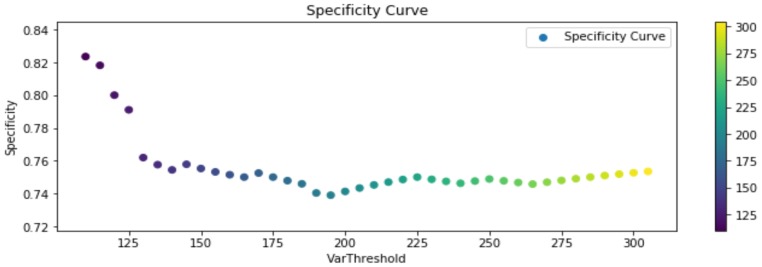
Specificity True Negative (TN)/(FP + TN) results.

**Figure 15:**
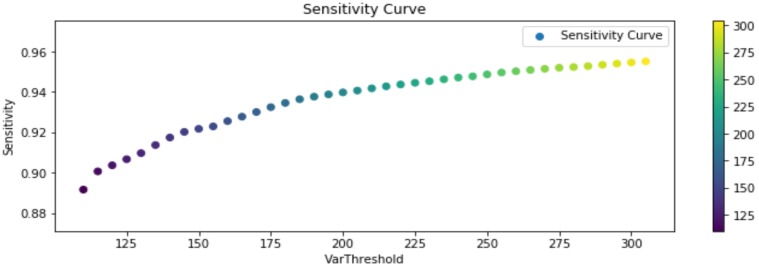
Sensitivity TP/(TP + False Negative (FN)) results.

The experimental results, considering the previous definitions, are shown in the following Figure 13, Figure 14 and Figure 15. Said figures were built by comparing events occurring in all the training routines in the dataset. Each player should trigger 20 events per routine, reaching a total of 180 events. Furthermore, due to the sensitivity of the segmentation stage to noise, illumination changes, shadows, and occlusion, test were performed considering different segmentation thresholds, for representing changes that could affect future experiments in an open field (soccer field).

In Figure 13 it can be observed that the precision of the algorithm remains stable as the segmentation threshold changes, in average overcoming a 97.5%. On the other hand, in Figure 14 specificity (cyanhow many of the true negative footsteps in a contact region (target) events were correctly identified as negative footsteps in contact region) reaches in average a 75%, fundamentally because the number of true negatives over the total of events in the ground-truth is not higher than 8%. Therefore, a low quantity of false positives has a great influence in this measure. Sensitivity in average holds over 90%, increasing along with the segmentation threshold, therefore the F1-Score associated with this measures is 93.6%, which means a good percentage of correct identification and a low rate of false alarms, but certainly improvable.

From the achieved results, the few false positive and false negative situations emerged are mostly due to the difficulty on discerning the actual position of both feet in the training platform Y axis, in self-occlusion situations. For this reason, the algorithm errors occur generally when the player tries to reach the most distant targets with respect to the camera (targets 7, 8, and 9), as shown in Figure 11a. However, the reached precision percentage indicates that the automatic registration tool has a better performance than the manual acquisition. The previous result is given by the capability of the tool for detecting erroneous target situations according to each stimuli sequence or non target-reaching situations, which are extremely difficult to be performed by a human operator in real-time.

**Figure 16:**
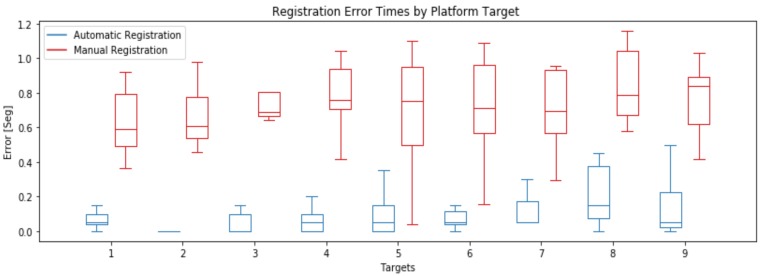
Boxplot of registration error times for Automatic and Manual Registration.

Let us suppose that manual registration has no error in target detection, that is to say, correctly detect all true positives and false positives target arrival times. The results of manual registration are compared against an automatic registration provided by our algorithm, both respect to the previous described ground-truth for each training video in the database. The results of this exercise are shown in Figure 16.

Results show that for any target the manual registration has a greater average time error, and a greater deviation than the automatic registration, while automatic registration has a greater deviation and average error times in targets that are located in platform back part, given the difficulty on measuring the position of both feet in the training platform Y axis (see Table 1).

Finally, it is important to remark that the player position is measured using only one frontal camera. Therefore, all the feet-crossing situations (self-occlusion) makes the gathering of the exact feet position very difficult, implying errors in footstep detection and target registration.

## 5. Conclusions

In this article we presented a new system for automatically registering the footstep interactions of players in the context of a new training method for reactive agility in soccer, Moya [8]. The main contribution is the development of the algorithm for automatic registration of footsteps in contact regions, along with classification of player training mistakes related with the stimuli sequence in the training routine, with the aim of reducing the subjectivity and errors produced by human operators. For this reason, the algorithm was evaluated, comparing with the performance of a human operator performing manual registration of footsteps in contact region. The experimental results shown high accuracy related with event detection.

Nevertheless, the performance of the algorithm can still be improved for discriminating the absence of footstep events. In this sense, the processing of occlusion situations could require further development, testing different camera resolutions and sample frequencies, as long as the real-time detection condition is kept.

As future work, it is planned to use a second camera arranged laterally to the platform (and synchronized with the frontal camera), to acquire more precise information about the feet positions in the platform Y axis. Given the second camera, it is proposed to extend the main algorithm for implementing two new Kalman filters (one for each foot in each frame for two cameras), for handling occlusion situations in a better way. For this purpose, new specific and more sensitive algorithm for handling multi-camera information must be developed.

## 6. Patents

The reactive agility training method presented in this article has the following international patent application:

Universidad Técnica Federico Santa María, Universidad de Playa Ancha de Ciencias de la Educación, Un Sistema y Método de Entrenamiento Perceptivo-Cognitivo para la Evaluación de Agilidad Reactiva en Deportistas Ante Diferentes Escenarios, PCT No: PCT/CL2019/050127, N. Ref:42/1937-1118-PCT, filed 02/12/2019.

## Figures and Tables

**Table 1:** Automatic Registration Average Error (AR-AE), Standard Deviation Error (AR-DE) and Manual Registration Average Error (MR-AE), Standard Deviation Error (MR-DE).

Target	1	2	3	4	5	6	7	8	9
AR-AE	0.07	0.01	0.05	0.07	0.09	0.11	0.13	0.2	0.15
MR-AE	0.64	0.66	0.78	0.75	0.72	0.71	0.68	0.84	0.76
AR-DE	0.07	0.01	0.05	0.07	0.09	0.11	0.13	0.2	0.15
MR-DE	0.18	0.19	0.19	0.22	0.25	0.28	0.24	0.22	0.21
